# Supplementary material for: Identification of photoperiod-induced specific miRNAs in the adrenal glands of Sunite sheep (Ovis aries)
Source: Front Vet Sci. 2022 Jul 22;9:888207. doi: 10.3389/fvets.2022.888207 (PMC9354845; doi:10.3389/fvets.2022.888207)
Supplement: Supplementary file 1 [file Data_Sheet_1.ZIP › Supplementary file/miRDeep2 file.zip!/miRDeep2 file/Novel_Structure/SPLP42A2/Novel_470.pdf]

Provisional ID	:	NC_019484.2_43468
Score total	:	91.4
Score for star read(s)	:	3.9
Score for read counts	:	84.2
Score for mfe	:	1.7
Score for randfold	:	1.6
Score for cons. seed	:	
Total read count	:	177
Mature read count	:	170
Loop read count	:	0
Star read count	:	7

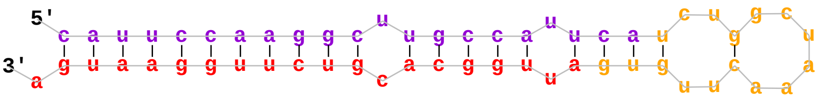

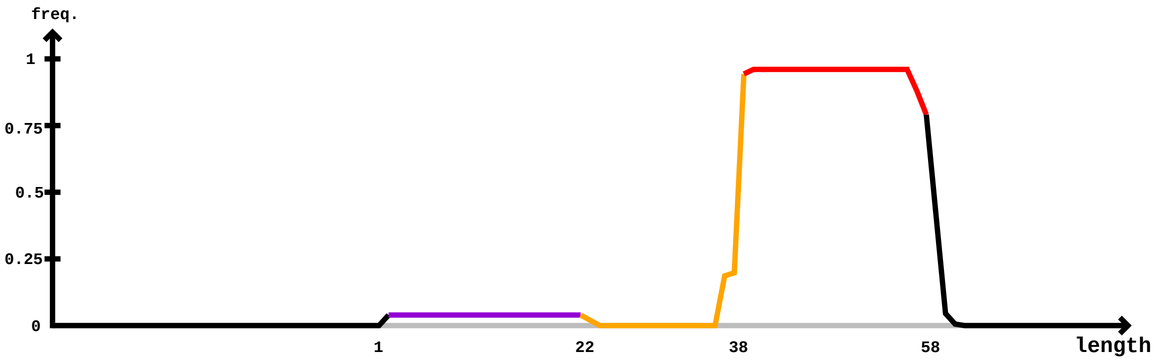


```
                                 Star                              Mature
5'- uugccaccuucagcgugucugugugcuguaccccauuccaaggcuugccauucaucuggcuaaacuugugauuggcacgucuuggaaugaaguaacguacaacaaugaug   -3'   obs
    uugccaccuucagcgugucugugugcuguaccccauuccaaggcuugccauucaucuggcuaaacuugugauuggcacgucuuggaaugaaguaacguacaacaaugaug         exp
    ..((........))..((((((((((..(((..(((((((((((.(((((.((((..(......)..)))).))))).)))))))))))..)))..)))).))).))).   reads   mm   sample
    .................................cauuccaaggcuugccauuca........................................................   7       0    seq
    ....................................................................ugauuggcacgucuuggaau......................   9       0    seq
    ....................................................................ugauuggcacgucuuggaaug.....................   3       0    seq
    ....................................................................ugauuggcacgucuuggaauga....................   19      0    seq
    ....................................................................ugauuggcacgucuuggaaugaa...................   1       0    seq
    ....................................................................ugauuggcacgucuuggaaugaag..................   1       0    seq
    .....................................................................gauuggcacgucuuggaauga....................   1       0    seq
    .....................................................................gauuggcacgucuuggaaugaa...................   1       0    seq
    ......................................................................auuggcacgucuuggaau......................   5       0    seq
    ......................................................................auuggcacgucuuggaaug.....................   13      0    seq
    ......................................................................auuggcacgucuuggaauga....................   80      0    seq
    ......................................................................auuggcacgucuuggaaugaa...................   27      0    seq
    ......................................................................auuggcacgucuuggaaugaag..................   6       0    seq
    ......................................................................auuggcacgucuuggaaugaagu.................   1       0    seq
    .......................................................................uuggcacgucuuggaauga....................   3       0    seq
```
